# Calcium-dependent signalling in B-cell lymphomas

**DOI:** 10.1038/s41388-021-02025-8

**Published:** 2021-10-08

**Authors:** Fedor Berditchevski, Eanna Fennell, Paul G. Murray

**Affiliations:** 1 Institute of Cancer and Genomic Sciences, The University of Birmingham, Edgbaston, Birmingham B15 2TT UK; 2 Health Research Institute, University of Limerick, Castletroy, Limerick, V94 T9PX Ireland; 3 Institute of Immunology and Immunotherapy, University of Birmingham, Birmingham, B15 2TT UK

**Keywords:** Haematological cancer, Cell signalling

## Abstract

Induced waves of calcium fluxes initiate multiple signalling pathways that play an important role in the differentiation and maturation of B-cells. Finely tuned transient Ca^+2^ fluxes from the endoplasmic reticulum in response to B-cell receptor (BCR) or chemokine receptor activation are followed by more sustained calcium influxes from the extracellular environment and contribute to the mechanisms responsible for the proliferation of B-cells, their migration within lymphoid organs and their differentiation. Dysregulation of these well-balanced mechanisms in B-cell lymphomas results in uncontrolled cell proliferation and resistance to apoptosis. Consequently, several cytotoxic drugs (and anti-proliferative compounds) used in standard chemotherapy regimens for the treatment of people with lymphoma target calcium-dependent pathways. Furthermore, ~10% of lymphoma associated mutations are found in genes with functions in calcium-dependent signalling, including those affecting B-cell receptor signalling pathways. In this review, we provide an overview of the Ca^2+^-dependent signalling network and outline the contribution of its key components to B cell lymphomagenesis. We also consider how the oncogenic Epstein-Barr virus, which is causally linked to the pathogenesis of a number of B-cell lymphomas, can modify Ca^2+^-dependent signalling.

## Mature B-cell differentiation

Around 90% of all B-cell neoplasms are derived from mature B cells [1], i.e., cells that have undergone rearrangement of their immunoglobulin (Ig) heavy chain and light chain genes in the bone marrow, express surface B-cell receptor (BCR) and have been selected against autoreactivity. The mature B-cells that emerge from the bone marrow are known as naive B cells. These cells co-express the BCR as IgM and IgD molecules and will become activated if their BCR recognises its cognate antigen. Thereafter, the antigen-activated B cells migrate into B cell follicles and establish a germinal centre (GC). In the GC, the B-cells proliferate and activate several key processes that include somatic hypermutation (SHM), which introduces mutations in the Ig heavy and light chain variable region genes to improve the affinity of the BCR, and class switch recombination (CSR) in which the isotype of the Ig heavy chain is changed to either IgG, IgA, or IgE [2]. Only a fraction of antigen-specific B cells survive the GC reaction and emerge as either plasma cells or memory B cells. Most B-cell lymphomas arise from cells that have been through a GC reaction, reflecting the increased risk of mutation in cells undergoing SHM and CSR.

## Activation of Ca^2+^-dependent signalling pathways in B cells

Changes in the intracellular calcium concentration in B cells are tightly controlled by coordinated signals initiated by cell-surface receptors (e.g., BCR, chemokine receptors) and by various calcium channels and antiporters.

*Signalling via the BCR*. Initiation of calcium-dependent signalling has best been characterised in the context of BCR activation. The binding of antigen to the BCR results in activation of the associated cytoplasmic tyrosine kinases (Syk and Btk), that, in turn, phosphorylate and activate phospholipase C gamma 2 (PLCγ2). PLCγ2 generates inositol trisphosphate (IP3) that binds to the IP3 receptors (IP3R) and induces the first wave of Ca^2+^ influx to the cytoplasm from the endoplasmic reticulum (ER). Calcium depletion from the ER stores is sensed by the ER-associated stromal interaction molecule 1 (STIM1), that is translocated to the junction between the ER and plasma membrane and activates calcium release‐activated channels (CRAC) - Orai1/2. This leads to the second wave of calcium influx to the cytoplasm and subsequent repletion of the ER stores. Several cell surface molecules control the BCR-induced calcium signalling in B cells [3]. For example, activation of Btk, an upstream regulator of PLCγ2, is also controlled by CD19-associated phosphatidylinositol-tris-kinase (PI3K), that facilitates the recruitment of Btk to the plasma membrane [4]. By contrast, BCR-induced phosphorylation of inhibitory Fc-receptors (FcγRIIB) and CD22, a member of the Siglec family of sialic acid receptors, recruit SHP-1 tyrosine phosphatase, that negatively regulates the upstream activators of PLCγ2 [5].

*Signalling via GPCRs*. Several chemokines and bioactive lipids, acting through G-protein-coupled receptors (GPCRs), control calcium waves in B cells [6]. Activation of GPCRs can either feed into BCR-induced Ca^2+^ fluxes via the Btk-PLCγ2 signalling axis [7] or via G-protein-associated PLCβs [8]. GPCRs can also act directly on the IP3R by activating cyclic AMP-dependent protein kinase A [9].

*Calcium channels in signalling*. In addition to CRAC-Orai-STIM complexes, B cells express non-store-operated calcium channels [10]. While their role in Ca^2+^-dependent signalling pathways and in the differentiation of mammalian B-cells remains largely unknown, experiments involving the avian B-cell line (DT40) demonstrated that TRPC3 and TRPC7, two diacylglycerol-activated TRP channels, may contribute to BCR-induced influx of extracellular calcium [11].

## Origin of mature B-cell lymphomas

The classification of the mature B-cell lymphomas into distinct entities is partly based on similarity to their normal B-cell counterparts and assumes that the malignant cells retain many characteristics of the normal cells. For example, Burkitt lymphoma (BL) expresses markers that are characteristic of normal germinal centre B-cells (e.g., BCL6 and CD10) [12]. Chronic lymphocytic leukaemia and small lymphocytic lymphoma (CLL/SLL) are characterized by the clonal expansion of CD5 + B cells. In many cases, the transformed cells remain dependent on the expression of a functional BCR; such tumours include BL, diffuse large B-cell lymphoma (DLBCL), mantle-cell lymphoma (MCL), CLL/SLL and marginal zone lymphoma (MZL). In some cases, there is evidence of aberrant BCR signalling driven in part by genetic alterations. For example, the two major ‘cell of origin’ subtypes of DLBCL, known as the activated B-cell (ABC) and germinal centre B-cell (GCB) forms, show strikingly different mechanisms of aberrant B-cell receptor (BCR) activation. In ABC-DLBCL, so-called chronic active BCR signalling activates both the nuclear factor-κB (NF-κB) and PI3K/AKT pathways [13]. This is a consequence of BCR cross-linking induced by self-antigens expressed on the same or adjacent cells, combined with sustained signalling resulting from pathway mutations, for example in CD79B [14]. In contrast, only the PI3K/AKT pathway is activated downstream of the BCR in GCB-DLBCL and signalling is antigen-independent and referred to as ‘tonic’ [15, 16]. The degree of mutation of the genes encoding the immunoglobulin heavy chain clonotype of the BCR is among the most robust prognostic tools for CLL/SLL, dividing patients with ‘unmutated’ CLL (U-CLL) as having more aggressive disease outcomes, or so-called ‘mutated’ CLL (M-CLL) as having more indolent disease course [17]. On the other hand, some mature B-cell lymphomas, for example, classical Hodgkin lymphoma (cHL) and primary mediastinal B cell lymphoma (PMBCL), are characterised by the loss of BCR functions. This can be a consequence of either so called ‘crippling’ mutations in the immunoglobulin-coding genes, epigenetic silencing of key BCR components or their transcriptional down-regulation, for example, by the Epstein-Barr virus (EBV)-encoded latent membrane proteins- 1 and 2 A [18–20].

While in BCR-expressing lymphomas receptor crosslinking is likely to provide the most powerful and sustained signal leading to activation of Ca^2+^-dependent pathways, engagement of several other surface receptors also feeds into calcium haemostasis. Lectin binding to DC-SIGN induces persistent activation of PLCγ2 in follicular lymphoma cells [21]. Activation of CXCR4 in DLBCL cells induces both transient (PLC-dependent) and sustained (STIM-ORAI-1-dependent) calcium influx [22]. Binding of Wnt5 to its Frizzled-5 receptor-induced Ca^2+^-dependent activation of NFAT and NF-κB in BL cells [23]. In BCR-independent lymphomas, calcium homeostasis is regulated by a number of other surface receptors. For example, ligation of the CD30 receptor expressed on malignant cells induces Ca^2+^ influx in cHL [24]. Similarly, stimulation of cysteinyl leukotrienes receptors (CysLTR1) induces strong calcium signalling in the malignant cells of both cHL and PMBCL [25, 26].

## Calcium-binding enzymes in B-cell lymphomas

**Fig. 1:**
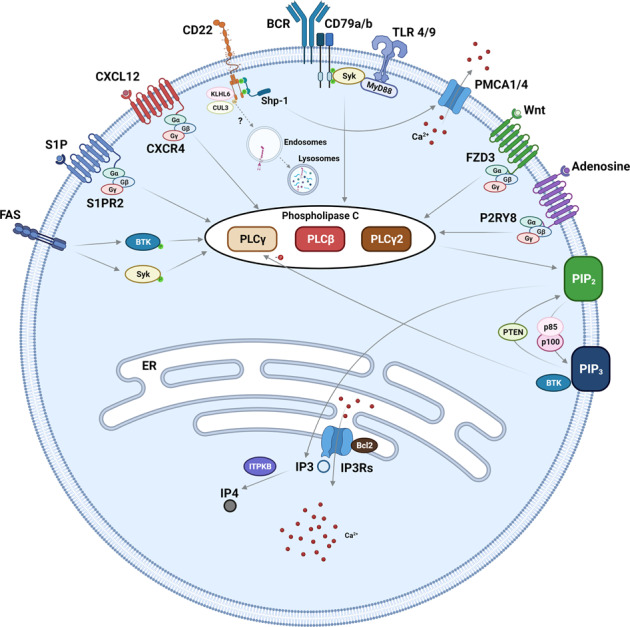
Major Ca^2+^-dependent pathways that could be affected by genes mutated in B-cell lymphomas (Table 1). Activation of surface BCR complex (i.e., membrane immunoglobulin (mIg) associated with covalently linked CD79a-CD79b heterodimer) and other surface receptors (e.g., TLRs, CXCR4, and FAS) by their ligands results in PLC-dependent hydrolysis of phosphatidylinositol bisphosphate (PIP2) and generation of inositol triphosphate (IP3). PLCs are activated either by Syk and Btk tyrosine kinases which induced tyrosine phosphorylation of the protein (e.g., PLCγ2) or via binding to Gβγ subunits of trimeric GTP-binding proteins (PLCβ) that are coupled to cell surface receptors (CXCR4, FZD3, P2RY8, and S1PR2). IP3-bound receptors (IP3Rs) on the endoplasmic reticulum (ER) induce release Ca^2+^ from the ER leading to activation of various cytoplasmic enzymes (Fig. 2). CD22 forms a tripartite complex with protein tyrosine phosphatase non-receptor type-6 (Shp-1) and plasma membrane Ca^2+^‐ATPase (PMCA) that regulates Ca^2+^ efflux pathway. Kelch-like protein 6 (KLHL6) functions as an adaptor for Cullin-3 (CUL3), an E3 ubiquitin ligase that regulates internalisation (and possibly degradation) of CD22 thus negatively affecting the contribution of the protein to calcium homeostasis. Phosphoinositide 3-kinases (PI3Ks, p85/p100) and Phosphatidylinositol 3,4,5-trisphosphate 3-phosphatase (PTEN) control interconversion of PIP2 and phosphatidylinositol trisphosphate (PIP3) on the plasma membrane. Binding to PIP3 enhances enzymatic activity of Btk resulting in increased Btk-dependent phosphorylation of PLCγ.

There are hundreds of calcium-binding proteins in cells whose activity could be affected by induced Ca^2+^ waves in B cells. Genetic profiling of B cell lymphomas has identified that a significant proportion of mutations occur in genes with functions in calcium-dependent signalling (Fig. 1, Table 1). Importantly, some of the reported mutations have been shown to enhance calcium-dependent homeostasis in model lymphoma cell lines [27, 28]. Here, we will only discuss Ca^2+^-dependent enzymes with established links to B-cell lymphomagenesis.

**Table 1:** Frequencies of mutations in genes involved in Ca^2+^-dependent signalling in B-cell lymphomas.

GENE	Protein	CLL/SLL	DLBCL	MCL	FL	BL	MZCL	cHL
**BTK**	BTK, Bruton Tyrosine Kinase	4.0	1.6	4.2	1.2	–	1.0	–
**CD79A**	B-Cell Antigen Receptor Complex-Associated Protein alpha Chain	0.2	2.8	–	1.2	–	0.5	4.8
**CD79B**	B-Cell Antigen Receptor Complex-Associated Protein beta Chain	0.3	9.6	–	1.4	4.7	1.8	1.3
**CXCR4**	C-X-C Motif Chemokine Receptor 4	1.4	2.3	–	2.2	9.7	3.5	–
**FAS**	Fas Cell Surface Death Receptor	0.4	6.2	4.9	12.5	–	22.8	1.3
**S1PR2**	S1PR2, Sphingosine-1-Phosphate Receptor 2	0.7	4.0	–	–	8.8	–	–
**PLCG2**	PLCγ2, Phospholipase gamma 2	2.0	0.8	3.8	–	12.9	1.1	–
**ITPR2**	Inositol 1,4,5-Trisphosphate Receptor Type 2	2.8	2.2	9.1	3.6	0.5	–	3.8
**ITPR3**	Inositol 1,4,5-Trisphosphate Receptor Type 3	0.5	1.4	–	3.6	–	–	3.8
**ITPKB**	Inositol-Trisphosphate 3-Kinase B	1.7	5.4	2.4	1.8	–	–	13.9
**FZD3**	FZD3, Frizzled Class Receptor 3	0.6	0.3	–	1.8	9.3	4.0	–
**P2RY8**	Purinergic Receptor P2Y8	0.2	2.3	–	5.1	1.5	–	–
**GNAI2**	G Protein Subunit Alpha I2	0.1	0.6	–	5.5	13.9	4.0	–
**KLHL6**	Kelch Like Family Member 6	2.5	5.4	–	–	1.0	1.1	–
**PTEN**	PTEN, phosphatidylinositol-3,4,5-trisphosphate 3-phosphatase	0.6	2.9	3.7	2.0	9.7	1.5	–
**PIK3R1**	p85A, Phosphoinositide-3-Kinase Regulatory Subunit 1	0.5	1.5	–	1.2	9.3	1.0	4.5
**BCL2**	B-Cell Lymphoma 2	2.9	18.8	1.9	36.5	0.5	3.5	–
**MYD88**	MYD88 Innate Immune Signal Transduction Adaptor	2.7	17.8	–	–	1.2	6.6	–

Data for the mutations were obtained from the catalogue of somatic mutations in cancer (COSMIC) database (cancer.sanger.ac.uk/cosmic).

## Protein kinase C (PKC) enzymes

The family of PKC is composed of 9 isoforms including a subgroup of Ca^2+^-dependent enzymes: PKCα, PKCβI/II, and PKCγ. While PKCγ is not expressed in B-cells, both PKCα and PKCβ have been shown to play important roles in B-cell differentiation [29]. Although the proliferation of B cells was not affected in PKCα-deficient mice, there was diminished antigen-specific IgM to IgG2a and IgG2b switch in these animals [30]. Experiments involving PKCβ-knockout and PKCβ-transgenic animals have revealed a signalling link between BCR-dependent activation of Btk and PKCβ [31–33]. An early study using PKCβ-knockout mice demonstrated that although BCR-induced signalling is suppressed in these animals, PKCβ also functions as a Btk-inhibitor in a negative feedback mechanism [33]. Specifically, PKCβ-induced phosphorylation of Btk inhibited the recruitment of the enzyme to the plasma membrane and its subsequent activation. Further experiments using cells from PKCβ-deficient mice demonstrated that PKCβ regulates the BCR-dependent metabolic switch to glycolysis in naive B-cells [34]. In addition, PKCβ is involved in the BCR-induced activation of NF-κB by phosphorylating CARMA1/CARD11, an important component of the CARMA1/Bcl10/Malt1 (CBM) complex, which functions as an upstream activator of the NF-κB pathway [35]. Finally, PKCβ isoforms acting downstream of BCR and Orai1 suppress the apoptotic signalling pathway by stimulating the expression of Bcl-2 and Bcl-xL on the one hand [36, 37], and inhibiting the assembly of the death-inducing signalling complex on the other [38].

Earlier reports demonstrated that high expression of PKCβ was associated with poor survival of DLBCL patients [39–41]. In addition, recurrent mutations in PRKCB (gene encoding PKCβ) has been observed in follicular lymphomаs [42]. Given its role in different BCR-induced signalling pathways, it is not surprising that knockdown and pharmacological inhibition of PKCβ suppresses chronic BCR-signalling in the ABC-subtype of DLBCL [43] and cell proliferation [37].

## Calpains

Calpains belong to a family of intracellular Ca^+2^-dependent cysteine proteases that are implicated in a variety of vital biological processes [44]. Activation of calpains downstream of BCR has been linked to B-cell clonal deletion and establishing the B-cell repertoire [45]. The role of calpains in the pathogenesis of B-cell lymphomas has not been systematically investigated. Studies involving two BL cell lines demonstrated that a calpain inhibitor II (CPI-2) triggers rapid apoptosis [46]. Conversely, BCR activation in WEHI-231 cells, a murine B-cell lymphoma line, induced calpain-dependent cleavage of Caspase-7 leading to apoptosis [47]. In another study, inhibition of calpains decreased the activity of NF-κB1 (p50) homodimers leading to downregulation of the expression of Bcl-2, one of the key anti-apoptotic proteins [48]. On the other hand, calpain-dependent cleavage of Bax, a pro-apoptotic member of the Bcl-2 family, may represent a critical initiation step in arsenic sulfide-induced apoptosis of human DLBCL cell lines [49]. In addition to the apoptosis-related signalling network, calpains have been implicated in proteolytic cleavage of Myc thus generating Myc-nick, a truncated protein lacking the C-terminal portion of c-Myc [50]. Myc-nick, that is present in precancerous bone marrow-derived pre-B cells along with full-length Myc in the Eμ − myc mouse model of Myc-driven lymphoma [51], was shown to bind microtubules and recruits acetyltransferases to promote α-tubulin acetylation and microtubule stabilization [50]. Finally, calpains were shown to act on Btk [52] and IP3R3 [53], important components of the BCR-dependent signalling network.

## Calcineurin

Calcineurin (CaN) is a ubiquitously expressed serine/threonine phosphatase that is activated by calcium and calmodulin, an abundant calcium-sensing cytoplasmic protein [54] (see below). BCR-induced transient release of calcium from internal stores, and sustained influx of extracellular calcium were shown to activate CaN [55]. Early experiments using EBV-positive and EBV-negative lymphoma cell lines suggested that CaN is involved in BCR-induced apoptosis involving activation of caspases-2 and -3 [56]. Subsequent studies demonstrated that in BL cell lines, BCR-induced rapid activation of CaN resulted in the nuclear translocation of NFATc2, a member of the family of nuclear factor of activated T-cells (NFAT) transcription factors, followed by the increased expression of pro‐apoptotic molecule TR3/Nur77 and initiation of the apoptotic programme [57]. The importance of the CaN-NFAT signalling axis in lymphomagenesis is also supported by the observation that CaN-dependent nuclear localisation of NFATc1 was seen in ~70% of BL and ~30% of DLBCL [58]. Although not investigated in B-cell lymphomas, some other CaN substrates have been linked to lymphomagenesis. Increased accumulation of the transcription factor FOXO3 in the nucleus facilitates apoptosis of DLBCL cells treated with ibrutinib, a specific Btk inhibitor [59]. Importantly, an earlier study demonstrated that dephosphorylation of FOXO3 in cells expressing high levels of CaN is accompanied by the nuclear translocation of FOXO3 [60]. Transcription factor EB (TFEB) has been identified in a genome-wide CRISPR screen for genes regulating apilimod-induced cytotoxicity in B-cell non-Hodgkin lymphomas [61]. Apilimod, an antiproliferative compound specifically targeting PIKfyve lipid kinase, induces rapid dephosphorylation of TFEB and protein translocation to the nucleus. While the underlying molecular pathways have not been illustrated in this report, TFEB has been described as a CaN substrate in other cell types [54].

## Calcium/Calmodulin kinases

**Fig. 2:**
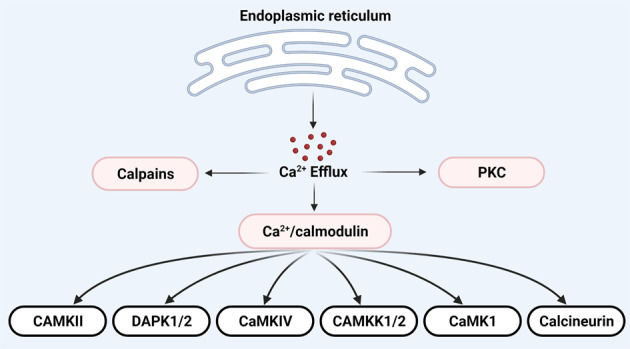
Intracellular enzymes activated by calcium. Elevations in intracellular Ca^2+^ result in activation of calmodulin-independent and calmodulin-dependent enzymes.

Ca^2^^+^ /calmodulin (CaM)-dependent protein kinases are activated after they interact with calcium-bound calmodulin (Fig. 2). Whilst several CaM kinases are expressed in normal B cells (https://www.proteinatlas.org/), their role in normal B-cell differentiation remains completely unknown. Reduced mRNA expression of death-associated kinase 1 (DAPK1) due to promoter hypermethylation was observed in DLBCL, FL and cHL [62, 63]. Furthermore, hypermethylation of the DAPK1 promoter is associated with more aggressive disease and poor outcome in CLL/SLL, DLBCL and FL [64–66]. Experiments involving model B-cell lymphoma cell lines demonstrated CaMKK2-dependent phosphorylation of AMPK may contribute to the induction of autophagy and apoptosis [67]. On the other hand, B-cell activating factor (BAFF) – induced proliferation and survival of normal B cells and BL cell line involved CaMKII-dependent phosphorylation of Akt and activation of the mTOR signalling pathway [68].

## The Epstein-Barr virus and Ca^2+^ signalling

EBV, a gamma human herpesvirus, is an aetiological agent in the pathogenesis of a number of cancers of B-cell origin [69]. In latently infected B cells, the EBV genome can express distinct latency programs [70–72]. The latency III program or the ‘growth program’ consisting of six EBV nuclear antigens (EBNA−1, −2, −3A, −3B, −3C, and −LP) and three latent membrane proteins (LMP−1, −2A, and −2B), is expressed in newly infected B cells, and in most cases of EBV-positive DLBCL [73]. Latency II or the ‘default program’ consisting of EBNA1, LMP1, LMP2A, and LMP2B, is expressed in EBV-infected germinal centre B cells and II is expressed in all cases of EBV-positive cHL [74–76]. Latency I, in which protein expression is limited to only one protein, EBNA1, is characteristic of dividing EBV-infected memory B cells and most cases of EBV-positive BL [70, 71]. Latently infected B cells can also enter the ‘lytic cycle’ which ultimately leads to the production of new virions and cell death.

## The latent membrane proteins of EBV are major modulators of calcium-regulated signalling pathways in EBV-infected cells

The latent membrane proteins, LMP1 and LMP2A, are expressed in the latency III and latency II programmes, and play a crucial role in calcium-regulated signalling pathways in EBV-infected cells. In turn, calcium signalling has profound effects on EBV life cycle, favouring virus persistence and oncogenesis.

LMP1 is a constitutively active CD40 homologue that can activate a number of signalling pathways, including NF-κB, JAK/STAT, AP-1, and PI3K/AKT signalling [77–79]. LMP1 expression in Latency II is thought to provide the CD40-like signals required to enable EBV-infected B cells to survive a germinal centre reaction and to subsequently differentiate into plasma cells or memory B cells [80]. LMP1 has been shown to induce many of the features of the aberrant transcriptional programme characteristic of the tumour cells of cHL, including the down-regulation of BCR signalling components, and the increased expression of anti-apoptotic genes such as BCL2 and BFL-1 [20, 81]. It has been reported that the CaM-dependent protein kinase II (CaMKII), that is activated by LMP1, is crucial for the ability of LMP1 to induce NF-κB signalling [82]. CaMKII interacts with interleukin-1 receptor kinase (IRAK1) and this is required for LMP1 induced CaMKII activation. CaMKII directly phosphorylates p65/RelA leading to p65/p50 or p65/p52 mediated transactivation [82]. LMP1 has been shown to increase calcium flux in B cells [83] and it is suggested that LMP1 might activate CaMKII by modulating calcium flux [82]. Specifically, EBV infection of BL cell lines was shown to increase expression of the high Ca^2+^ affinity SERCA2, and decrease expression of the low Ca^2+^ affinity, SERCA3, resulting in an increase in the amount of Ca^2+^ in the lumen of the ER [84]. LMP1 was shown to be responsible for the decrease in SERCA3 expression [83]. LMP1 also activates CaMKIV expression in B cells [84]. Both NFATc1 and NFATc2 induce lytic EBV gene expression when combined with activated CaMKIV [85]. The increased activity of NFAT transcription factors is reported to account for the increased viral lytic cycle activity in B-cells infected with Type 2, compared with Type 1, EBV [85]. In keeping with a crucial role for calcium signalling in regulating the viral lytic cycle, calcineurin inhibitors have been shown to block EBV entry to the lytic cycle following BCR stimulation [86]. LMP2A mimics some of the functions of BCR signalling, for example, by activating ERK/MAPK and PI-3K pathways which is likely to be important for the survival of infected germinal centre B cells [87–89]. At the same time, it was shown that LMP2A can sequester key components of the BCR signalling complex, including Syk and Lyn, thereby partially blocking BCR signalling in infected cells to prevent induction of the virus replicative cycle and thereby maintaining latency [90, 91]. However, it has subsequently been shown that in the absence of other stimuli, LMP2A can activate the lytic cycle [92]. We and others have shown that while some of the downstream consequences of BCR activation and LMP2A expression do overlap, there are notable differences [93, 94]. Thus, LMP2A concordantly regulates the tyrosine phosphorylation of PI-3-kinase, Syk, and the Ca^2+^ initiation complex (comprising BLNK, BTK, and PLCγ2), resulting in oscillatory Ca^2^+ fluxes similar to those observed after BCR stimulation [94–96].

## Ca^2+^ homeostasis and therapeutic implications

Cytosolic Ca^2+^ concentration is affected by a number of chemotherapy drugs, some of which are commonly used for treatment of lymphoma patients [97]. Whilst their specific molecular targets and affected Ca^2+^-dependent pathways have been extensively investigated in the context of non-haematopoietic cells (normal and malignant) [97–100], there are only a limited number of studies that address the effect of the drugs on calcium homeostasis in B cell lymphomas.

Humanised anti-CD20 mAbs are routinely used for the treatment of B-cell lymphomas [101]. Targeting CD20 with rituximab (RTX) or Obinutuzumab induces both transient and sustained (Orai1–dependent) influx of Ca^2+^ to the cytoplasm and activation of Ca-dependent signalling pathways linked to the antibody-induced cell death [102, 103]. It has been proposed that targeting CD20 with therapeutic antibodies induces CD95(FAS)-dependent and CD95-independent apoptosis [102, 103]. Sensitivity to RTX-based chemotherapy was shown to be dependent on the expression levels of Cav1.2, a L-type voltage-gated calcium channel and treatment with a channel agonist markedly increased the sensitivity of DLBCL cell lines to RTX [104].

The R-CHOP therapy (a combination of RTX with cyclophosphamide, doxorubicin, vincristine and prednisolone) is a standard treatment for DLBCL patients. However, ~40% of the patients treated with R-CHOP are either resistant to the initial treatment or will subsequently relapse [105]. Elevated expression of a number of calcium-binding proteins has been associated with resistance to R-CHOP treatment in DLBCL patients. TRPM4 is a calcium-dependent monovalent cation channel that transports K^+^ and Na^+^ into cells, which, in turn, regulate intracellular Ca^2+^ homeostasis [106]. TRPM4 is overexpressed in ABC-DLBCL, and correlates with poorer overall survival of patients treated with R-CHOP [107]. In another study, R-CHOP-resistant DLBCL cell lines expressed high levels of Sorcin [108], a cytosolic multifunctional calcium-binding protein that is also highly expressed in DLBCL samples when compared with normal lymphoid tissues [109].

Ibrutinib has proven to be effective in the treatment of both indolent and aggressive B-cell lymphoma [110]. Ibrutinib inhibits BCR-induced calcium flux in normal B cells [111]. Gain of function mutations in the gene encoding PLCγ2 [112] are thought to be one of the major factors responsible for resistance to Ibrutinib in patients with MCL [113].

Bortezomib, a reversible proteasome inhibitor is approved for the treatment of relapsed or refractory MCL. The cytotoxic effect of bortezomib on established MCL cell lines and MCL-initiating cells was enhanced in the presence of L-type calcium channels inhibitor potentially through a more potent inhibitory effect on the activation of the NF-κB pathway [114].

## Future directions

There remain significant gaps in our understanding of how Ca^2+^-dependent signalling contributes to lymphomagenesis. While we need to expand our knowledge of how calcium-dependent enzymes discussed in this review regulates cellular responses in different B-cell lymphomas, a systematic “omics-based” approaches may be necessary to dissect the exact nature of Ca^2+^-centred signalling networks and how they operate in normal and malignant B-cells. These studies should include other cytoplasmic and secretory proteins whose activity is dependent on calcium (e.g., cyclic nucleotide phosphodiesterase (PDE), adenylyl cyclase, S100, transglutaminase). For example, the role of various plasma membrane calcium pumps and channels in controlling basal intracellular levels of Ca^2+^ levels requires investigation, as does how changes in the expression of these proteins affect steady-state activities of calcium-dependent enzymes. The results of these experiments may be particularly relevant for lymphoma patients with disrupted calcium haemostasis leading to hypercalcemia, that is associated with shorter progression-free and overall survival [115, 116].

Communication between the malignant B-cells and other cell types in the tumour microenvironment is also a critical factor in B-cell lymphomagenesis [117]. Although not investigated in the context of B-cell lymphomas, the contribution of CREB- and NF-κB-induced secretion of cytokines and chemokines downstream of calcium-dependent pathways are well documented in other tumour types. At the post-transcriptional level, calcium-binding proteins in the ER (i.e., calnexin and calreticulin) are well placed to control early steps of the secretory pathway [118]. Finally, calcium-dependent signalling pathways are known to control the composition of vesicles secreted by cancerous cells [119], thus affecting the behaviour of other cells in the immediate tumour microenvironment.

Whilst novel therapeutic approaches are beginning to emerge, most lymphoma patients continue to be treated with standard chemotherapy with or without CD20-targeting antibodies. A better understanding of how commonly used chemotherapy drugs affect the expression and function of key players within calcium-centred signalling networks may provide a solid basis for further optimisation of the current treatment protocols and the development of new therapeutic strategies.
